# Cold tolerance strategies of the fall armyworm, *Spodoptera frugiperda* (Smith) (Lepidoptera: Noctuidae)

**DOI:** 10.1038/s41598-022-08174-4

**Published:** 2022-03-08

**Authors:** Mohammad Vatanparast, Youngjin Park

**Affiliations:** Plant Quarantine Technology Center, Animal and Plant Quarantine Agency, Gimcheon, 39660 Republic of Korea

**Keywords:** Molecular biology, Physiology, Zoology

## Abstract

The fall armyworm (FAW), *Spodoptera frugiperda*, is native to the tropical and subtropical areas of the American continent and is one of the world's most destructive insect pests and invaded Africa and spread to most of Asia in two years. Glycerol is generally used as a cryoprotectant for overwintering insects in cold areas. In many studies, the increase in glycerol as a main rapid cold hardening (RCH) factor and enhancing the supercooling point was revealed at low temperatures. There are two genes, including glycerol-3-phosphate dehydrogenase (GPDH) and glycerol kinase (GK), that were identified as being associated with the glycerol synthesis pathway. In this study, one GPDH and two GK sequences (GK1 and GK2) were extracted from FAW transcriptome analysis. RNA interference (RNAi) specific to *GPDH* or *GK1* and *GK2* exhibited a significant down-regulation at the mRNA level as well as a reduction in survival rate when the RNAi-treated of FAW larvae post a RCH treatment. Following a cold period, an increase in glycerol accumulation was detected utilizing high-pressure liquid chromatography and colorimetric analysis of glycerol quantity in RCH treated hemolymph of FAW larvae. This research suggests that GPDH and GK isozymes are linked to the production of a high quantity of glycerol as an RCH factor, and glycerol as main cryoprotectant plays an important role in survival throughout the cold period in this quarantine pest studied.

## Introduction

The fall armyworm (FAW), *Spodoptera frugiperda* (Smith), is native to the American continent's tropical and subtropical regions^1^. It is a polyphagous insect, and due to its wide host range, it is one of the most dangerous pests affecting tropical annual crops^2,3^. They are usually composed of two genetically distinct strains, such as rice (R-strain) and corn (C-strain)^4–6^. FAW was reported in a number of Southeast Asian countries in 2018 and 2019, including India, Thailand, Myanmar, China, Japan, the Philippines, Indonesia, and most recently, Australia. The first invaded populations of FAW in South Korea were genetically confirmed using a mitochondrial cytochrome oxidase subunit I (COI) gene in 2019^7^. The presence of ideal climatic conditions for FAW in many parts of Africa and Asia, as well as an abundance of suitable host plants, indicates that the pest can produce many generations in a single season, and that the pest is likely to become endemic^8^. The chance of FAW spreading would be greatly increased by its long-distance migration. The first confirmation of the invasion of FAW in Yunnan Province (western area) of China was documented on January 11, 2019. FAW had spread to most provinces in southern China by May 2019^9^. In reality, due to low temperatures, FAW can only successfully breed in the summer and cannot survive the winter in most areas of mainland China, Japan, and the Korean, so these areas will need to be reinvaded on an annual basis^10,11^. The East Asian migration area includes the Japanese Islands, Korea, and eastern China. The geographical place, ecological climate, and climatic conditions of these areas are all intertwined. Many seasonal pests, such as rice plant hoppers (*Nilaparvata lugens* (Stål), *Sogatella furcifera* (Horváth), and *Laodelphax striatellus* (Fallén)) and the oriental armyworm, *Mythimna separate* (Walker), can fly from China to Japan and the Korea^12–14^. Now that FAW has made its way into Southeast Asia and southern China and southern Korea, the pest has a better possibility of invading Japan and Korea^9^. FAW has posed a significant threat to local corn and other crop production, as well as food security. Modeling the insect's rate of expansion and future potential migratory range using a trajectory analytical method and meteorological data during five years (2014–2018) revealed a very high probability that FAW will annually invade Korea, potentially causing a substantial decrease in agricultural productivity.

Since FAW do not diapause, they migrate to areas with better environmental conditions^4^. Even though Sparks estimated the minimum temperature for survival to be 10 °C^15^, it was discovered in 1979 that temperatures below 13 °C at FAW overwintering sites do not enable larvae and pupae to survive^1^. After exposing all stages of FAW to low temperatures for three hours, it was discovered that the egg was the most resistant, with a 30% survival rate at – 10 °C^16^. Temperature is a vital abiotic variable that influences organisms' geographic distribution and seasonal activity patterns^1–5^ and it has a big impact on pest biology, and abundance^17^. With behavioral avoidance, migration, diapause or in an extremely altered physiological state, insects escape extreme temperatures^18^. Since insect development takes place within a certain temperature, a change in temperature can affect the development rate, lifespan and ultimately survival of the insects^19^. Because of their poikilothermic nature, low temperatures act as a physical barrier preventing insects from expanding their habitats^20^. The insect’s survival capability is characterized as cold hardiness after exposure to low temperature levels. This procedure leads to the development of particular compounds known as cryoprotectants, which are polyols and sugars^21^. Insects withstand cold temperatures by holding their body fluids liquid below their normal melting point (freeze-intolerant) or by avoiding ice formation in their tissues (freeze-tolerance)^22–24^. The primary strategy of freeze-intolerant insects is to avoid exposure to lethal temperatures. In contrast, freeze-tolerance insects are able to overcome freezing by employing a variety of mechanisms, such as reducing ice formation in cells or delaying ice formation^21,25^. Another strategy, termed ‘supercooling’ is focused on the ability of insects to be cooled until spontaneous ice nucleation happens within their body fluids. The supercooling point (SCP) is the temperature at which body water spontaneously freezes^24,26^. While body fluid cools below its freezing point during the supercooling state, no crystallization occurs. However, in many situations, death is likely to happen at temperatures far above the SCP^27,28^. Insects may quickly change their response to low temperatures, either by preventing chilling injury or by modifying their behavior, a process known as rapid cold hardening (RCH) that it is associated with chemical changes in hemolymph composition to increase polyols^29^. Exposure to 5 °C for 6 h in *Spodoptera exigua* (Hübner) caused a major RCH in all developmental stages, from egg to adult, which was accompanied by a strong increase in glycerol titers in hemolymph^30^. RCH for 2 h at 5 °C of the newly-emerged adult of five coleopteran grain-related species substantially increased the survival at different temperatures below zero as compared to the non-acclimated period^31^.

According to a recent study on the cold hardiness of invasive FAW species in China, pupae and older larvae have a much higher survival rate than eggs and younger larvae, and FAW can live in some southern areas of China's subtropical zone during the winter based on the SCPs of developmental stages at low temperatures and China's climatic regionalization. Supercooling capacity of *S. exigua*^32^ and its RCH^33,34^ allows it to live at low temperatures in temperate areas during the winter. Based on high pressure liquid chromatography (HPLC) analysis of glycerol titers in response to pre-exposure to a low temperature, it was demonstrated that glycerol is a key cryoprotectant in RCH in *S. exigua*^30^.

Because of FAW’s dispersal ability and high spreading efficiency, as well as its large reproductive capacity and wide host plant range, the pest is likely to become one of the most important migratory insect pests in South Korea that already categorized as a quarantine pest. FAW may increase cryoprotectant contents in hemolymph, such as glycerol, and may be able to endure cold seasons, but its high spreading efficiency and dispersal ability may also assist it in migrating from harsh to moderate environments. In this study we hypothesized that FAW use RCH and glycerol as an associated factor to survive to low temperatures. To investigate the function of glycerol, we used RNA interference (RNAi) to knock down genes involved in glycerol biosynthesis and then examined the intensity of RCH and glycerol accumulation.

## Results

### Glycerol content in plasma in response to low temperatures and exposure time

**Figure 1:**
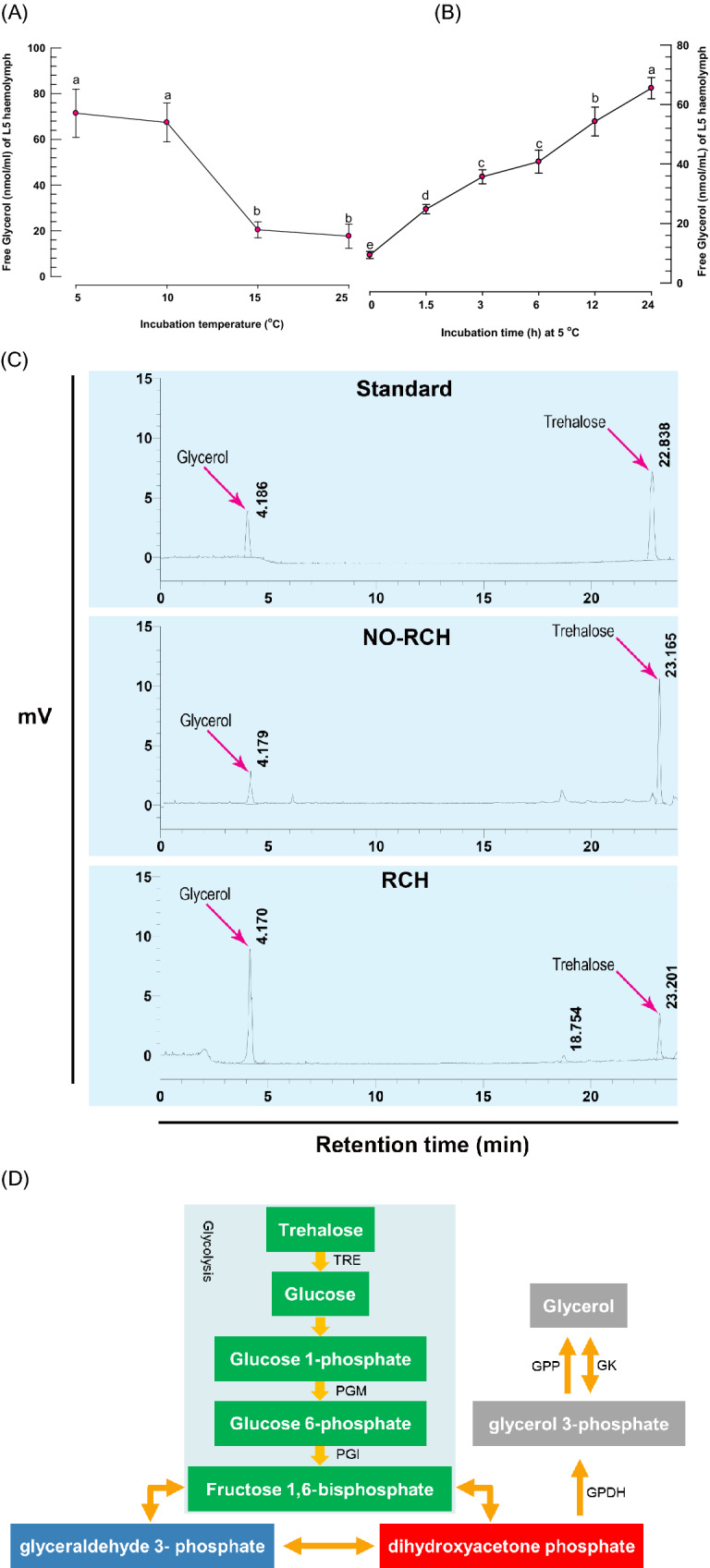
Measurement of free glycerol in plasma of L5 of *Spodoptera frugiperda* (**A**) at different temperature when the larvae incubated for 24 h. (**B**) Effect of exposure time on glycerol content of plasma when the larvae were incubated at 10 °C. Each treatment was replicated three times with 10 larvae per replication. Different letters indicate significant differences among means at (Type I error = 0.05, LSD test). (**C**) Chromatograms of hemolymph extracted from larvae exposed to 5 °C for 24 h. (**D**) A putative glycerol production pathway. Glycerol is formed by catabolizing glucose to dihydroxyacetone-3-phosphate (DHAP), which is then reduced to glycerol-3-phosphate (G3P). TRE, PGM, PGI, and GPP represent for trehalase, phosphoglucomutase, phosphoglucoisomerase, and glycerol‐3‐phosphate phosphatase, respectively.

When fifth instar larvae of FAW were incubated at low temperatures (5 and 10 °C) compared to higher temperatures, their glycerol content increased more than threefold (15 and 20 °C) (Fig. 1A). The exposure period was also linked to an increase in the amount of glycerol in plasma, with the most glycerol found after 24 h of incubation (Fig. 1B). The high glycerol level indicated that it is a major component of plasma after cold stress.

### Molecular architecture of glycerol biosynthesis genes

**Figure 2:**
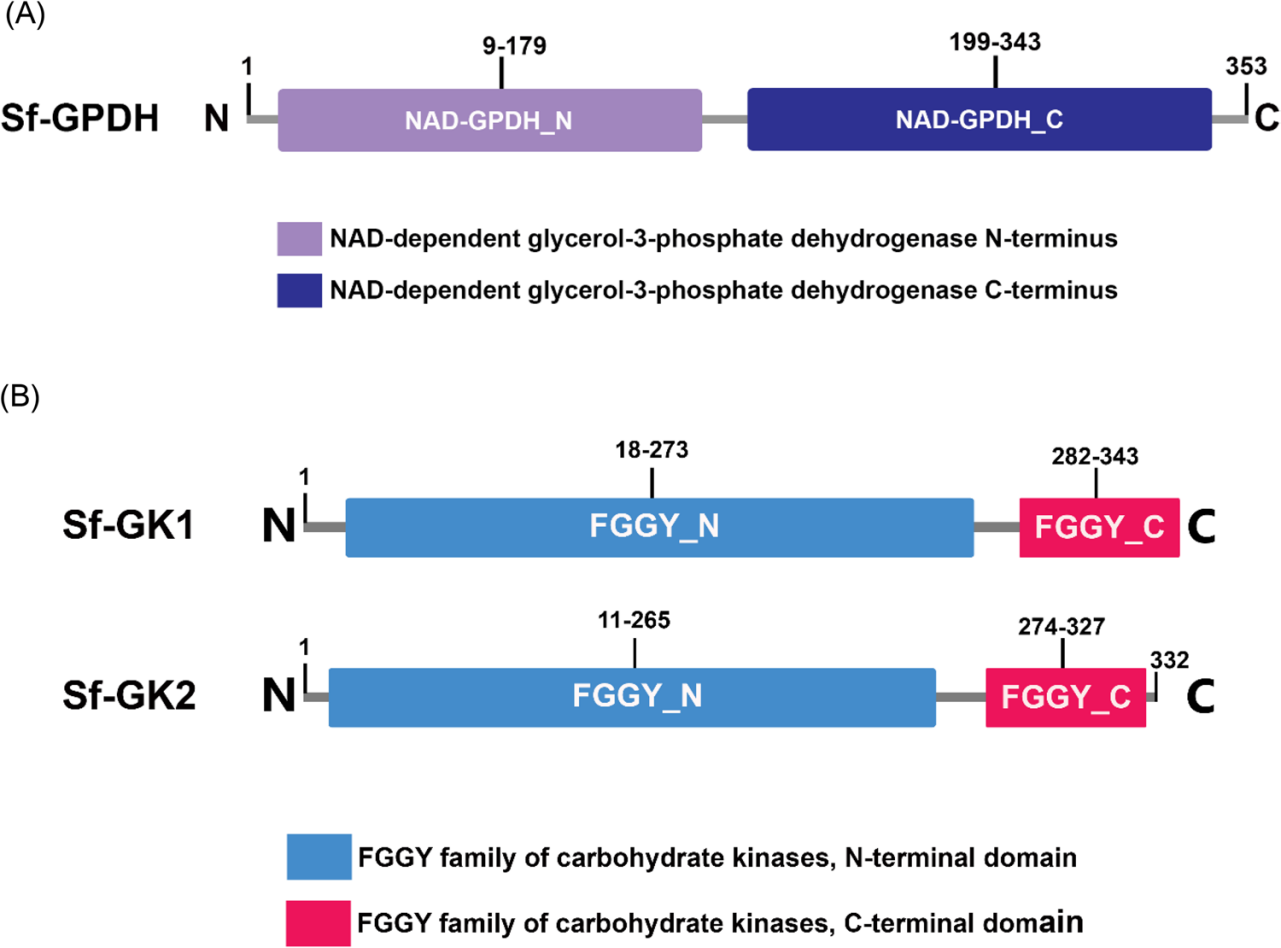
Protein domain analysis of glycerol biosynthesis genes of *Spodoptera frugiperda*. (**A**) Prediction of signature motifs of Sf-GPDH. (**B**) Prediction of signature motifs of Sf-GK1 and Sf-GK2. The functional domains were predicted using the NCBI conserved domain database and the EMBL-EBI HMMER database.

**Figure 3:**
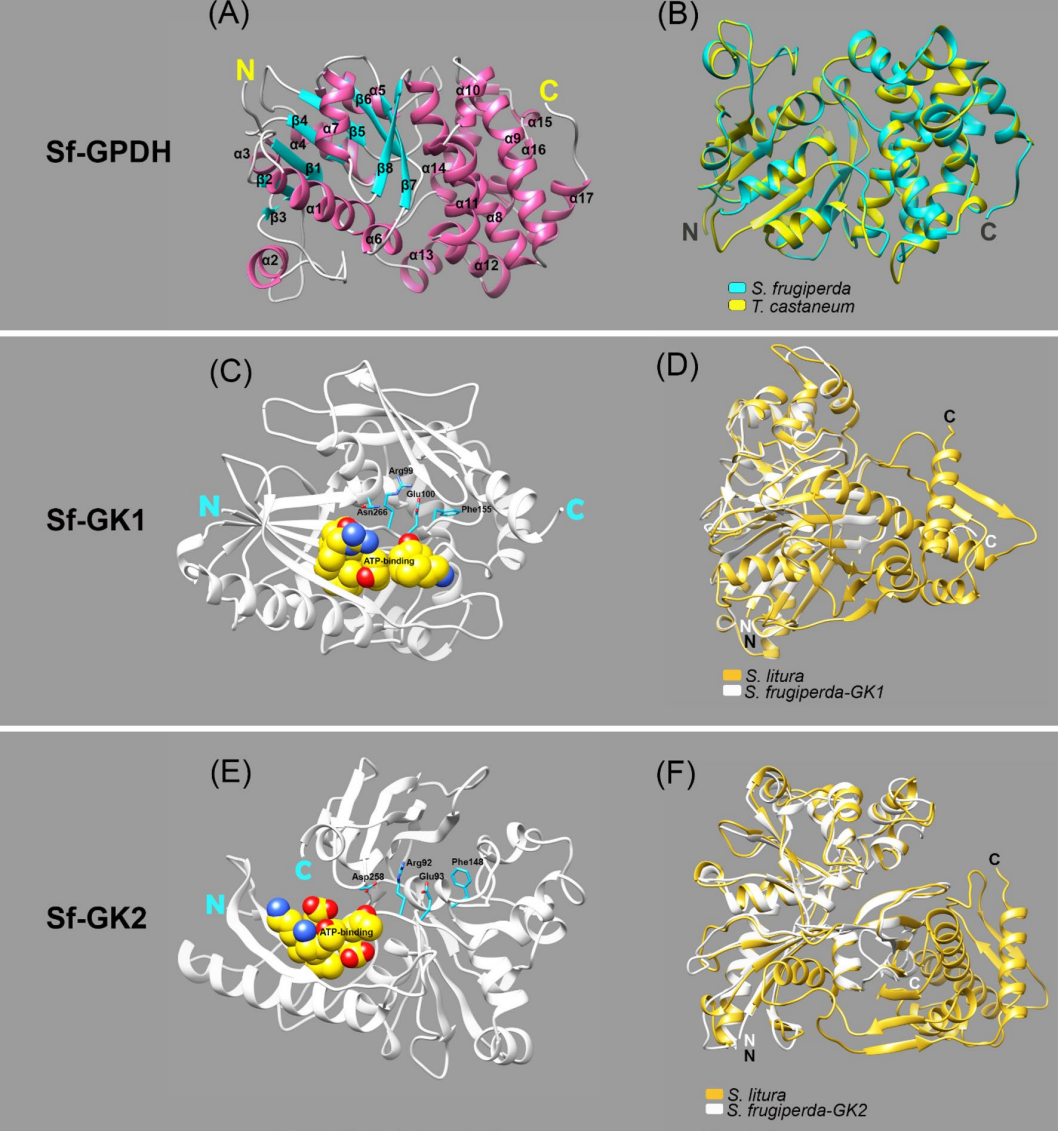
Three-dimensional analysis of glycerol biosynthesis genes of *Spodoptera frugiperda*. (**A**, **C**, and **E**) The functional domains of Sf-GPDH, Sf-GK1, and Sf-GK2 were demonstrated, respectively. (**B**, **D**, and **F**) the Sf-GPDH, Sf-GK1, and Sf-GK2 proteins respectively, were compared with same protein from another well-known insect, including *Tribolium castaneum* and *Spodoptera litura*. Blue and pink region in (**A**) indicate beta sheet and alpha helices, respectively. In (**C** and **E**), the glycerol binding residues were indicated with blue atoms as well as yellow part that showing ATP-binding domains. N and C are an abbreviation for N-terminus and C-terminus of amino acid sequences. These models were made using SWISS-model web database. Three dimensional constructs were made using Chimera, version 1.13.1.

**Figure 4:**
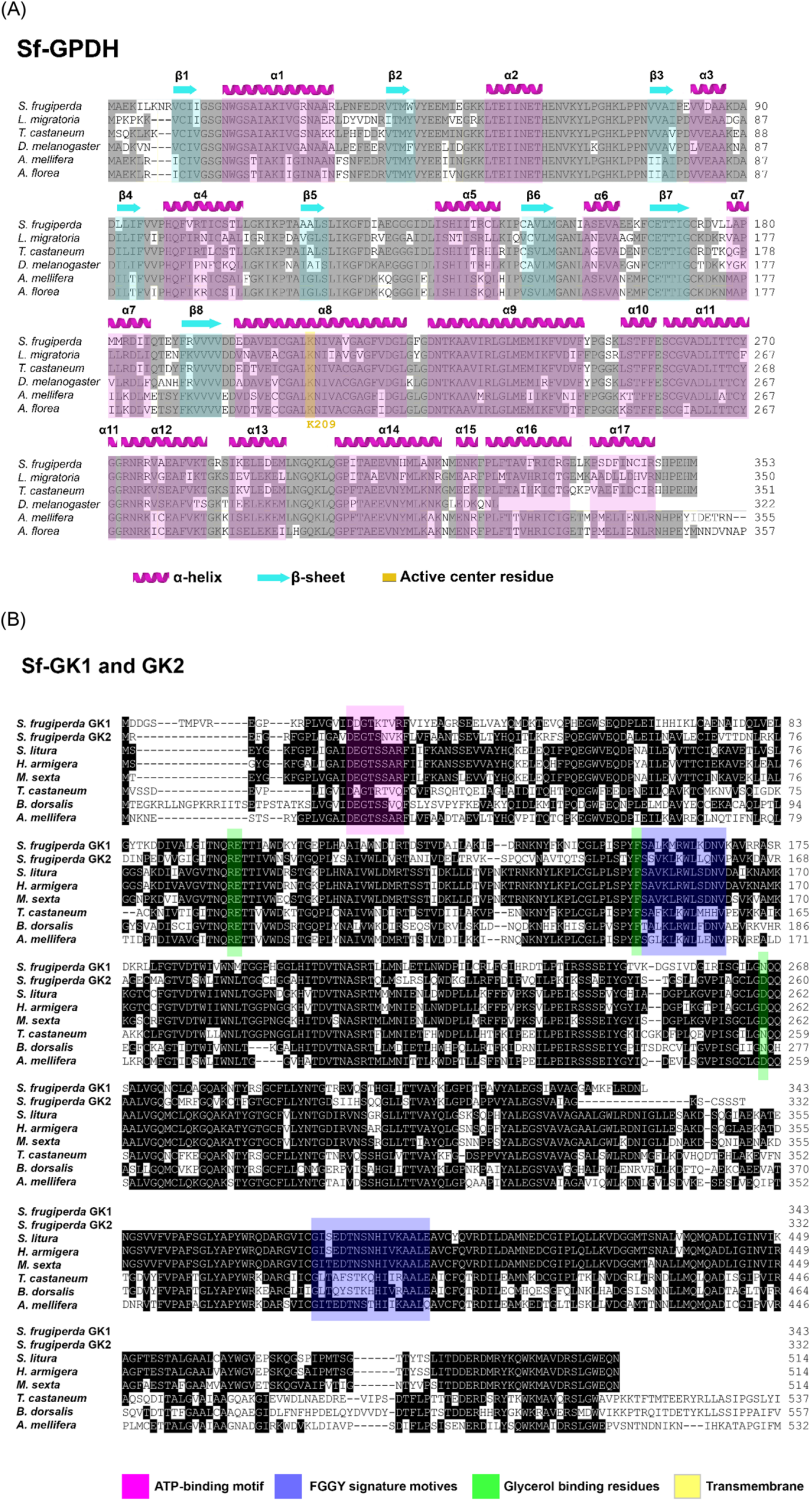
Alignment of amino acid sequences of glycerol biosynthesis genes including (**A**) glycerol-3-phosphate dehydrogenase (GPDH) and (**B**) two glycerol kinases (GK1 and GK2) of *Spodoptera frugiperda* with other well-known insect. Sequence alignment used Clustal W program of MegAlign (DNASTAR, Version 7.0). The abbreviation explanation and GenBank accession numbers of sPLA2 amino acids are listed in Table S2.

**Figure 5:**
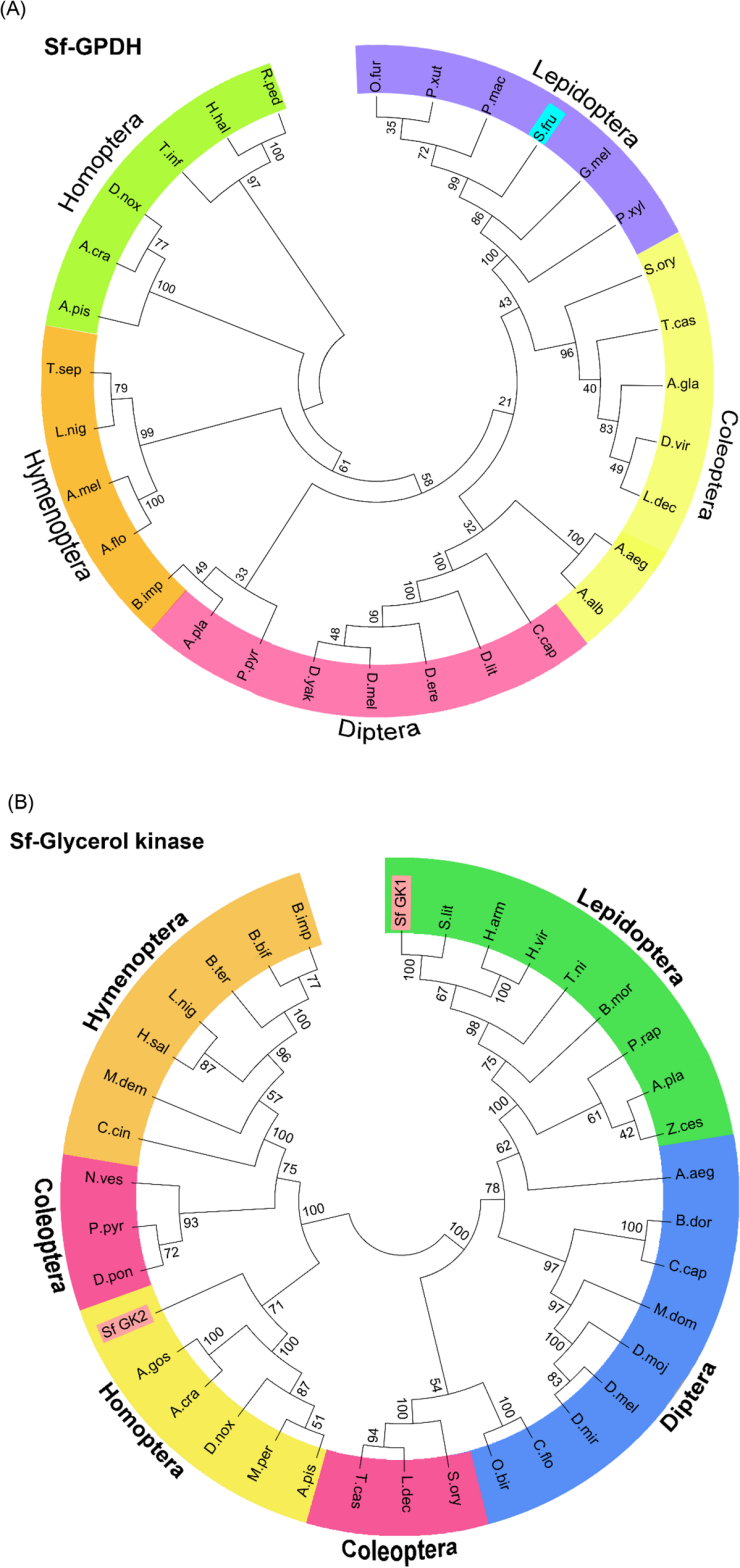
Phylogenetic analysis of glycerol biosynthesis genes including (**A**) glycerol-3-phosphate dehydrogenase (GPDH) and (**B**) two glycerol kinases (GK1 and GK2) of *Spodoptera frugiperda* with other insect species from different orders. The analysis was performed using MEGA6.06. Each node contains bootstrap value after 1000 replications to support branching and clustering. Accession numbers and the abbreviation explanation are shown in Table S2.

To explain the increase in glycerol content, we attempted to identify the enzymes involved in glycerol biosynthesis (Fig. 1C). Based on a previous study^30^ we chose dihydroxyacetone-3-phosphate (DHAP) as a precursor of glycerol biosynthesis from glycolysis intermediates. The catalytic activity of GPDH and GK converts DHAP to glycerol (Fig. 1D). As a result, the GPDH and GK genes were predicted to be involved in the synthesis of glycerol, which is an important cryoprotectant in insects when temperatures are extremely low. The transcriptome of FAW (NCBI accession number: GSE175545) were used to determine full open reading frames (ORFs) of GPDH (Sf-GPDH) and GK (Sf-GK1 and Sf-GK2) of FAW. Sf-GPDH, Sf-GK1 and Sf-GK2 ORFs encode for 353, 343, and 332 amino acid residues, respectively. Sf-GPDH protein contains a bi-domain protein structure, as illustrated in Fig. 2A that it encoded NAD^+^-dependent GPDHs with an N-terminal NAD^+^-binding domain and a C-terminal NAD^+^-dependent GPDH domain. Both identified FAW glycerol kinases shared N-terminal (FGGY-N) and C- terminal (FGGY-C) domains, which are colored blue and red, respectively, as shown in Fig. 2B, confirming that the targeted proteins are members of the FGGY carbohydrate kinase family. Based on comparisons with other well-known insect proteins, the three-dimensional structures of Sf-GPDH, Sf-GK1, and Sf-GK2 proteins were predicted using the homology modeling method (Fig. 3). These findings indicated that the sequences of Sf-GPDH and two Sf-GKs closely matched the homologous templates on the server, indicating that these protein models were reliable. The GPDH domain structure of Sf-NAD^+^-binding revealed two key components: a spatially symmetric β-sheet core and multiple helices (α1–α17) wrapping on both sides of the β-sheet core. The bioinformatics analysis indicated that four functional amino acids including Arg99, Glu100, Phe155, and Asn266 in Sf-GK1 and Arg92, Glu93, Phe148, and Asp258 in Sf-GK2 which are as glycerol binding residues (Fig. 3C, E). Three-dimensional analysis indicates 66% homology of Sf-GPDH with *Tribolium castaneum* (Herbert) GPDH under 73% coverage. When the Sf-GK1 and Sf-GK2 were compared by *Spodoptera litura* (Fabricus) glycerol kinase, the homology was 45 and 48% under 54 and 59% coverage (Fig. 3D, F). In these two glycerol kinases, several amino acids were conserved including ATP-binding motif and FGGY signature motives (Figs. 3, 4). A phylogenetic analysis indicated that the Sf-GPDH and Sf-GK1 were clustered with lepidopteran insects quite distinct from other insect orders. However, interestingly Sf-GK2 was clustered with Homopteran insect (Fig. 5).

### Expression profile of glycerol biosynthesis genes and inducible expression in response to low temperature in FAW

**Figure 6:**
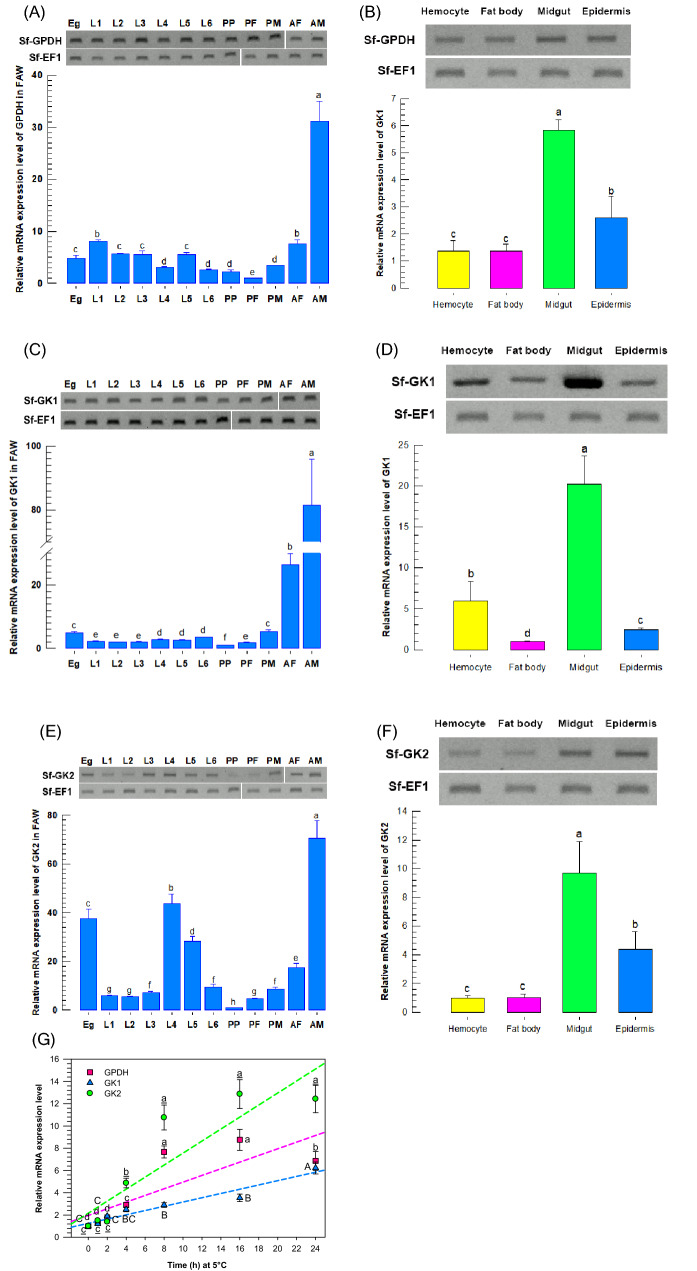
Expression analysis of glycerol biosynthesis genes from *Spodoptera frugiperda*. RT-PCR analysis of Sf-GPDH, Sf-GK1, and Sf-GK2 in different developmental stages (**A**, **C**, and **E**, respectively) and tissues (**B**, **D**, and **F**, respectively). The gels in (**A**, **C**, and **E**) were cropped from various gels and were cleared with vertical white space. The full-length gels are included in Supplementary Information (Fig. S2). *EF1* was used to validate cDNA integrity. Different developmental stages included Egg (‘Eg’), larval instars (ʻL1–L6ʼ), pre-pupa (‘PP’), pupa of female (ʻPFʼ), pupa of male (‘PM’), adult of female (ʻAFʼ) and adult of male (‘AM’). Different tissues included hemocyte, fat body, midgut, and epidermis. (**G**) RT-PCR analysis of Sf-GPDH, Sf-GK1 and Sf-GK2 expression at low temperature (5 °C) at different exposure time (h). Agarose gel (1%) was used for electrophoresis. Each treatment was replicated three times. Different letters above standard deviation bars indicate significant difference among means at Type I error = 0.05 (LSD test).

Three glycerol biosynthesis genes were expressed in FAW (Fig. 6). They were expressed from egg to adult in whole stages of development (Fig. 6A, C, E). In larval stage, they were expressed in different tissues such as hemocytes, fat body, midgut, and epidermis (Fig. 6B, D, F). However, their expression levels were varied among treatments during the developmental stages. All the three genes showed high expression level at adult stages of female insects. The highest expression level of all three genes was detected at midgut tissue. The expression levels of all three glycerol biosynthesis genes were inducible in response to low temperature (5 °C), and they showed a positive correlation with increasing incubation time. (Fig. 6G).

### Glycerol content is reduced by RNAi targeting glycerol biosynthesis genes following RCH

**Figure 7:**
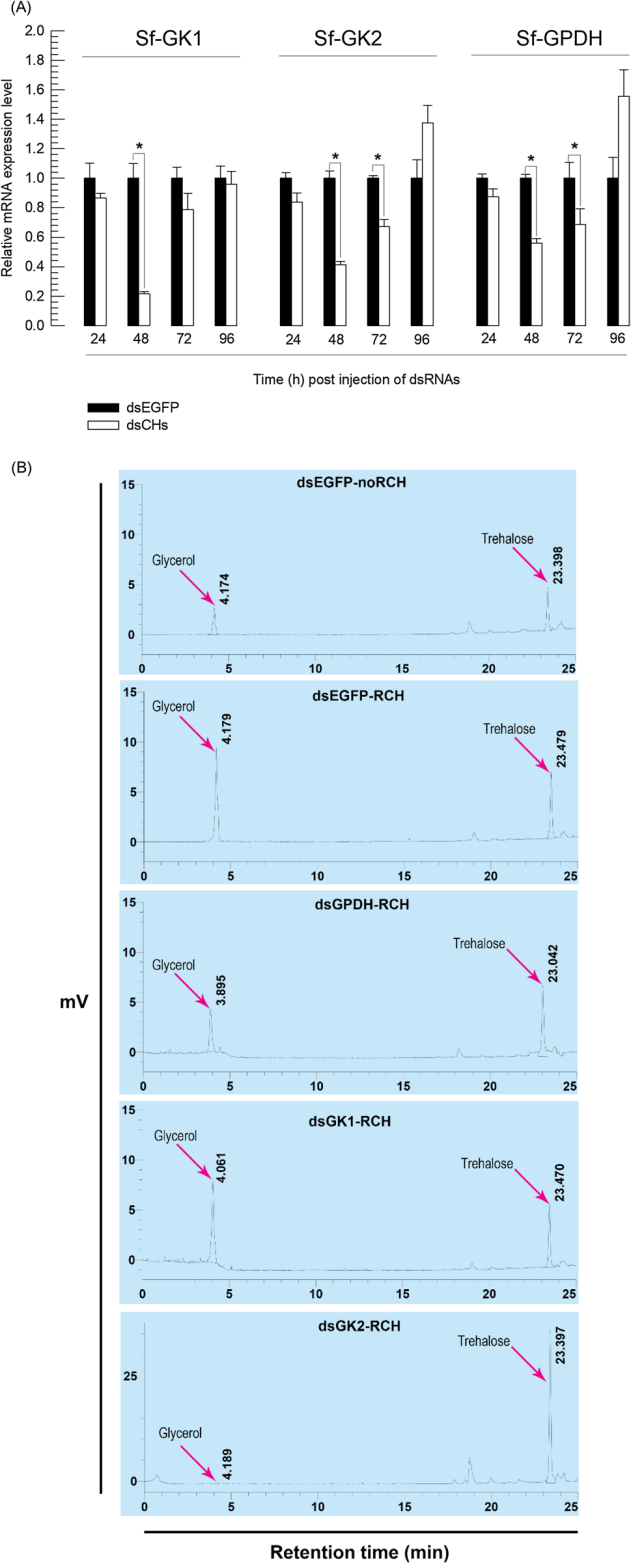

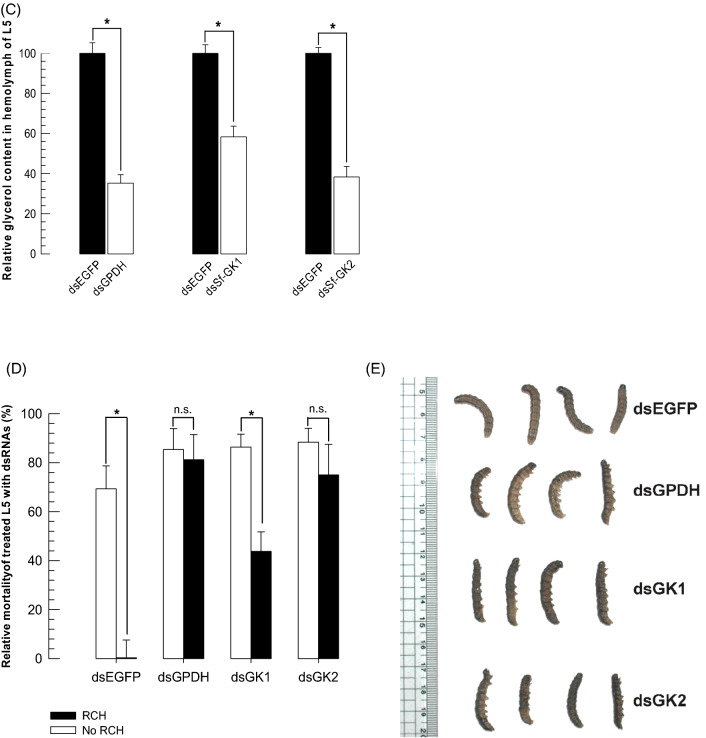
The effect of RNA interference (RNAi) specific to genes linked with glycerol biosynthesis, GPDH and GK of *Spodoptera frugiperda*. All dsRNAs specific to target glycerol biosynthesis genes were constructed at ~ 300–400 bp and injected to each L5 larva at 3 μg. Control RNAi (‘dsEGFP’) was injected with dsRNA specific to EGFP gene. (**A**) Quantitative real-time PCR to monitor changes in mRNA levels of Sf-GPDH, Sf-GK1, and Sf-GK2 after RNAi. *EF1* was used to validate cDNA integrity. (**B**) Chromatogram of HPLC that shows the effect of RNAi specific to glycerol biosynthesis genes, (48 h post dsRNAs injection) and RCH treatment (5 °C for 6 h), on glycerol content in hemolymph of fifth instar larvae. (**C**) Suppression of cold tolerance after RNAi treatment of either GPDH or GK1 or GK2. The glycerol content in hemolymph of fifth instar larvae was measured after 48 h of dsRNAs injection. (**D**, **E**) After RNAi injection (48 h post injection) and RCH treatment (5 °C for 6 h), the larvae were incubated at − 10 °C for 1 h and the mortality was recorded. Each treatment was replicated three times with 10 individuals per replication. Asterisks indicate significant difference between RCH and no RCH treatments (Type I error = 0.05, LSD test). n.s. means no significant difference.

RNAi was done on each glycerol biosynthesis gene (*Sf-GPDH*, *Sf-GK1*, and *Sf-GK2*) by injecting gene specific double-stranded RNAs (dsRNAs) into L5 larvae (Fig. 7). All three genes showed significant decreases (*P* < 0.05) with incubation time when one µg of dsRNA for each gene was injected into each larva. In all three genes, the strongest RNAi effect was observed at 48 h post injection, with a ⁓ 40–80 percent drop in mRNA expression levels (Fig. 7A).

RNAi downregulation of glycerol biosynthesis gene expression significantly suppressed glycerol amount (*P* < 0.05) in plasma at 48 h post-dsRNA injection after RCH treatment (Fig. 7B). The larvae treated with dsRNA for three genes had a basal amount of glycerol (29–35 mmol/mL), but control larvae (injected with dsRNA to enhanced green fluorescent protein (EGFP) gene) had approximately 73 mmol/mL glycerol (Fig. 7C). After RCH treatment and RNAi, the cryoprotectant(s) was monitored in hemolymph of fifth instar larvae using HPLC (Figs. 1C, 7B). Glycerol content significantly increased from 17.1 to 44.0 mM (Table 1) when the larvae were incubated at 5 °C. RNAi treatment larvae also showed a reduction in glycerol level when compare with control treatment (EGFP). Injection of dsGK2 resulted in a significant reduction in glycerol levels of more than seven times (6.08 mM) (Table 1, Fig. 7B).

**Table 1:** Change polyol content in *Spodoptera frugiperda* fifth instar hemolymph in response to exposure to 5 °C.

RNAi treatment groups	Glycerol (mM)/hemolymph of fifth instar	Trehalose (mM)/hemolymph of fifth instar
dsEGFP-noRCH	17.10 ± 0.98^d^*	4.42 ± 0.10^d^
dsEGFP-RCH	44.07 ± 0.95^a^	5.99 ± 0.13^c^
dsGPDH-RCH	22.58 ± 0.82^c^	6.89 ± 0.08^b^
dsGK1-RCH	40.36 ± 0.30^b^	5.70 ± 0.17^c^
dsGK2-RCH	6.08 ± 0.03^e^	21.53 ± 0.23^a^

Each treatment was replicated three times with 10 individuals per replication. Different superscript letters indicate significant difference between means for each polyol (Type I error = 0.05, LSD test).

*Different superscript letters indicate significant difference between means for each polyol (Type I error = 0.05, LSD test).

### RNAi of glycerol biosynthesis genes increases the mortality of treated larvae of FAW

Larvae at 48 h post-dsRNA injection did increase their mortality after RCH treatment (Fig. 7C, D). There was no significant difference in mortality between RCH and control (no RCH) treatment after RNAi of either Sf-GPDH or Sf-GK2, However the mortality decreased significantly when the larvae injected with dsRNA specific to Sf-GK1 after RCH treatment than to control (Fig. 7C).

### Following RCH treatment, the SCP increased

The effect of RCH on SCP was evaluated in all developmental stages including both sexes in pupal and adult stages (Table 1). Egg, first instar and pupal stages exhibited SCP at lowest temperature than other developmental stages. The data showed that supercooling capacity was unaffected by RCH treatment in egg, first and second instar, male pupae, and female adult whereas SCP temperature in the others was significantly reduced (Table 2). From these data, we found that RCH treatment is often accompanied by elevated SCPs. To investigate the involvement of glycerol biosynthesis genes in SCP, RNAi-treated larvae (L3 to L6) were incubated at RCH conditions and their SCP was assessed (Table 3). The SCPs of larvae injected with dsRNA specific to glycerol biosynthesis genes, specifically dsGPDH and dsGK2, were significantly lower than those of dsEGFP-injected larvae, suggesting that glycerol biosynthesis genes elevate their SCP by accumulating extracellular cryoprotectant including glycerol in their bodies. In compared to the dsEGFP treatment group, the SCPs of larvae injected with dsGK1 were significantly lower than to dsGPDH and dsGK2 injected larvae (Table 3).

**Table 2:** Changes in supercooling points of *Spodoptera frugiperda* after rapid cold hardening treatment (5 °C for 6 h).

Stage	RCH treatment	N	SCP (°C)
Egg	No	90	− 17.50 ± 0.56^a^*
	Yes	90	− 17.80 ± 0.35^a^
L1	No	90	− 17.20 ± 1.40^a^
	Yes	90	− 17.50 ± 0.64^a^
L2	No	60	− 14.60 ± 1.10^a^
	Yes	60	− 15.80 ± 0.85^a^
L3	No	10	− 10.62 ± 0.59^b^
	Yes	10	− 14.12 ± 0.73^a^
L4	No	5	− 12.15 ± 0.62^b^
	Yes	5	− 14.15 ± 0.45^a^
L5	No	5	− 8.58 ± 0.93^b^
	Yes	5	− 13.88 ± 0.48^a^
L6	No	5	− 8.35 ± 0.49^b^
	Yes	5	− 13.39 ± 0.45^a^
Pre-pupae	No	5	− 14.62 ± 0.67^b^
	Yes	5	− 16.19 ± 0.57^a^
Pupae (male)	No	5	− 18.47 ± 1.19^a^
	Yes	5	− 18.87 ± 0.53^a^
Pupae (female)	No	5	− 16.90 ± 0.35^b^
	Yes	5	− 18.20 ± 1.12^a^
Adult (male)	No	5	− 12.33 ± 1.20^b^
	Yes	5	− 14.15 ± 0.58^a^
Adult (female)	No	5	− 13.23 ± 1.52^a^
	Yes	5	− 14.53 ± 0.82^a^

Each treatment was replicated three times with different individuals per replication. Different superscript letters indicate significant difference between means for each SCP (Type I error = 0.05, LSD test).

*Different superscript letters indicate significant difference between means for each SCP (Type I error = 0.05, LSD test).

**Table 3:** Changes in supercooling points of *Spodoptera frugiperda* (third to sixth instar) after RNAi and rapid cold hardening treatment.

Stage	RCH treatment	N	SCP (°C)			
			dsEGFP	dsGPDH	dsGK1	dsGK2
L3	No	18	− 17.20 ± 0.53^a^*	− 12.30 ± 0.87^c^	− 15.76 ± 0.45^b^	− 8.65 ± 0.44^d^
	Yes	18	− 17.30 ± 0.51^a^	− 13.70 ± 0.73^b^	− 16.95 ± 0.34^a^	− 9.75 ± 0.61^c^
L4	No	18	− 16.80 ± 2.40^a^	− 10.24 ± 0.70^b^	− 16.21 ± 0.42^a^	− 7.88 ± 0.68^c^
	Yes	18	− 17.70 ± 0.62^a^	− 10.32 ± 1.12^b^	− 16.80 ± 1.10^a^	− 8.54 ± 0.48^c^
L5	No	18	− 14.10 ± 0.80^a^	− 8.76 ± 0.92^c^	− 12.76 ± 0.62^b^	− 7.36 ± 0.74^c^
	Yes	18	− 15.70 ± 1.85^a^	− 10.23 ± 0.65^b^	− 13.48 ± 0.74^a^	− 9.87 ± 0.56^c^
L6	No	18	− 11.82 ± 0.32^a^	− 7.94 ± 0.62^c^	− 9.54 ± 0.76^b^	− 7.24 ± 0.23^c^
	Yes	18	− 13.02 ± 0.52^a^	− 9.39 ± 0.45^b^	− 10.88 ± 0.33^b^	− 7.56 ± 0.36^c^

All dsRNAs specific to target glycerol biosynthesis genes were constructed at ~ 300–400 bp and injected to each L5 larva at 3 μg. Control RNAi (‘dsEGFP’) was injected with dsRNA specific to EGFP gene. The supercooling point was measured after RNAi injection (48 h post injection) and RCH treatment (5 °C for 6 h). Each treatment was replicated three times with 18 individuals per replication. Different superscript letters indicate significant difference between means for each SCP (Type I error = 0.05, LSD test).

*Different superscript letters indicate significant difference between means for each SCP (Type I error = 0.05, LSD test).

## Discussion

Many insect species can develop cold-hardiness well below freezing temperatures, and various features of insect cold-hardiness have been studied^23,35^. The most significant part of acclimatization for cold resistance is low temperature exposure^22,36^. Low-weight molecular molecules, often known as cryoprotectant, such as polyols and sugars, are produced during this procedure^21^. The most prevalent cryoprotectants include polyols (glycerol, sorbitol, and manitol), sugars (glucose, trehalose, and fructose), and amino acids^37–40^. High polyol concentrations not only lower the temperature at which an insect's body fluids crystallize but also stabilize the state of proteins, even when collected in relatively low concentrations^41^. Polyols regulate the amount of water accessible for freezing, which reduces the amount of cell dehydration caused by extracellular freezing. They protect biological membranes and proteins from freezing-induced dehydration by preserving their structures^41,42^. In the present work, the tolerance of FAW was analyzed by rapid cold hardening (RCH). In insects without diapause, RCH is especially important for overcoming lethal cold shock by rapidly increasing cold tolerance^20^. RCH has been induced in a variety of insects at temperatures ranging from 0 to 5 °C^30,43–47^. Glycerol production is divided into two distinct pathways, depending on the insect. In *Epiblema scudderiana* (Clemens), a moth belongs to Tortricidae family, polyol dehydrogenase catalyzes the reaction of glyceraldehyde with NADPH + H^+^ in one route^48^. The other pathway converts dihydroxyacetone-3-phosphate to glycerol via GPDH/GK (*S. exigua*)^30^. Identification of key genes associated with overwintering in *Anoplophora glabripennis* (Motschulsky) larva, a coleopteran species, using gene co-expression network analysis, was demonstrated that, fatty acid desaturase, glycerol phosphate dehydrogenase, glycerol kinase, and trehalose phosphate synthase were among the 15 genes implicated in the control of antifreeze protectants^49^. We studied on GPDH and GK genes expression to investigate the glycerol production pathway. In the FAW transcriptome, we discovered two GK isoforms and one GPDH isoform. It was discovered that both genes expressed and associated with glycerol biosynthesis pathway. The whole *Plutella xylostella* (Linnaeus) genome was used to predict four GKs and one GPDH^50^. The genome of FAW contains only one type of GPDH, indicating that it is a unigene with a conserved biological function in metabolism. Because we obtained these sequences from transcriptome data and there are likely no other endogenous genes of GPDH and GK, we believe our expression and functional analysis are associated with these isozymes. GPDH and both GK isoforms were discovered to be widely expressed in different studied tissues. As we know at low temperatures, most gene expression decreases^51^. However, in 5 °C, real-time PCR of cold-exposed larvae revealed that GPDH, GK1, and GK2 were expressed at relatively high levels (Fig. 6G). This suggests that these proteins are important for cold tolerance to the low temperature by RCH. As found in other insects^52,53^, cold tolerance rose as acclimatization time increased, which could be in line with our findings, that mRNA expression levels of analyzed genes increased as incubation time increased (Fig. 6G).

RNAi is a non-invasive way of delivering dsRNA into insects to knockdown specific gene expression^54–57^. We have shown that injecting RNAi is feasible and can suppress the transcription level of target genes in FAW larvae. Our system confirmed the effective knockdown of three genes at the mRNA expression level.

Their expression was knocked-down by specific dsRNAs associated with glycerol biosynthesis genes. In response to pre-exposure to a low temperature, this RNAi treatment reduced RCH and prevented glycerol accumulation. According to the RNAi experiments, sorbitol dehydrogenase, trehalose-6-phosphate synthase, and glycerol kinase are all involved in the overwintering stage of Chinese white pine larvae (*Dendroctonus armandi* (Tsai and Li))^58^. Glycerol phosphorylation, which is essential for glycerol consumption, is catalyzed by GK^59,60^. GK has a function in overwintering termination in *Hyalophora cecropia* (Linnaeus) eggs that accumulate glycerol by converting glycerol to glycerol-3-phosphate for other intermediary metabolism^61^. In *A. glabripennis* larvae, the gene expression level of glycerol kinase increased sharply at the midpoint of the overwintering stage, and then declined at the latter, which corresponded to the change in glycerol content. The findings suggest that glycerol kinase is involved in the synthesis of glycerol, which could help this insect adapt to low temperatures^49^.

Because RNAi targeting GK1 and GK2 significantly reduced glycerol accumulation in a 5 °C pretreatment, it was shown that both GKs catalyze the dephosphorylation of glycerol-3-phosphate to generate glycerol, as reported earlier in *S. exigua*, a near Noctuidae species to FAW^30,62^. However, it was discovered that GK2 has a greater effect on glycerol production based on RNAi data for mortality, glycerol accumulation, and HPLC results. The significant increase in Sf-GK2 expression vs Sf-GK1 shows that it has physiological significance in RCH, as evidenced by the RNAi functional study. *P. xylostella* GK1 showed a significant increase in expression in response to 5 °C exposure vs other three isozyme of GKs^50^. In *Bombyx mori* (Linnaeus), at least three GK isozymes have been discovered, but only one, GK3, appears to be connected to glycerol utilization^63^. The knockdown of the target genes Sf-GPDH, Sf-GK1, and Sf-GK2 not only reduces their transcription levels but also affects larval cold-tolerance capacity, leading to an increase in low-temperature mortality. The most obvious explanation for these findings is that these genes are necessary for overwintering larvae's cold tolerance. As there is no existing evidence of systemic spread in Lepidoptera^64^, we were unable to totally silence these three genes, however, the partial knockdown had a clear effect on low-temperature mortality.

The hemolymph polyol analysis revealed that trehalose was the primary blood sugar, with a concentration of 4.42 mmol^−1^ in hemolymph and a slight increase with low temperature exposure (5.99 mmol^−1^ after 6 h at 4 °C). Trehalose titers in insect hemolymph are relatively high in general, but very considerably between insects (ranging from 0.1 to 133 mmol^−1^)^30^. We detected 5.7 mM of trehalose following dsGK1-RCH treatment, whereas the titer increased considerably to 21.53 mM following dsGK2-RCH treatment. This may be a compensating effect of the glycerol depletion. However, we believe that in order to obtain more precise results, we need incorporate trehalose(s) (which catalyzes the conversion of trehalose to two glucose monomers) into our future studies.

In conclusion, due to a lack of a diapause mechanism, FAW cannot overwinter in area with a cold winter, despite the fact that they can disperse thousands of kilometers north during the growth season^65,66^. However, in this study, RNAi investigation of two types of important genes linked to glycerol production and their effects on glycerol accumulation and insect mortality in response to low temperature pre-exposure, revealed that glycerol is a substantial cryoprotectant in RCH in FAW. Increased glycerol concentrations may contribute to whole animal freeze tolerance by enhancing cell survival by freeze-tolerant. However, each cryoprotectant may have a distinct non-overlapping function and contribute to freeze tolerance through memchanisms distinct from those of others with different potency. In addition, the permeability of different tissues to cryoprotectants can be vary, affecting their ability to protect cells and this constituents. Supercooling data clearly demonstrated that FAW can endure very low temperatures, and as a key agricultural pest, it may be able to become one of most important migratory insect pests in Korea. To limit the impact of this pest, it is critical to create pest management strategies and detecting systems. In addition, more research on migration behavior is needed to predict source areas and migration times.

## Materials and methods

### Insect rearing, exposure temperatures and sample preparation

The larvae of FAW were obtained from Frontier Agriculture Sciences (Newark, DE, USA) and used F4 and F5 generations, which were maintained in laboratory. They were raised until pupation under laboratory-controlled conditions (26 °C, 70% RH, and a photoperiod of 14 h:10 h [L:D]). The larvae were fed an artificial diet (Newark, DE, USA) during their development^67^ and larval instar (L1-L5) were determined by head capsule sizes and molting times. The diet was changed every day for larvae. These larvae were grown in plastic containers with aerated lids measuring 40 × 20 × 15 cm. From the third instar onwards, larvae were reared separately to prevent cannibalism. This was carried out in Petri dishes (8.5 cm diameter).

### Bioinformatics to predict Glycerol synthase genes

The mRNA sequences for all genes were obtained from a previous study of transcriptome analysis of whole body of FAW using TruSeq RNA Sample Prep Kit v2 (Macrogen, Seoul, South Korea). The predicted amino acid sequences were aligned using Clustal W program of MegAlign (DNASTAR Version 7.0). The phylogenetic trees were constructed with Neighbor-joining method and Poisson correction model (1,000 bootstrap repetitions to support branching clusters) using MEGA 7.0 software (www.megasoftware.net). Conserved domains of glycerol synthase genes were predicted using NCBI Conserved Domain Database (www.ncbi.nlm.nih.gov/cdd). UCSF Chimera (https://www.cgl.ucsf.edu/chimera/) was used for protein motif analysis and making 3D structure.

### RNA extraction and RT-qPCR

RNAs samples were extracted from FAW larvae using Trizol reagent (Invitrogen, Carlsbad, CA, USA)^68^. After RNA extraction, it was resuspended in nuclease-free water and quantified using a spectrophotometer (NanoDrop, Thermo Scientific, Wilmington, DE, USA). cDNA was then synthesized from RNA (1 μg) using RT PreMix (Intron Biotechnology, Seoul, Korea) containing *oligo dT* primer according to the manufacturer's instruction. All quantitative PCRs (qPCRs) in this study were determined using a Real time PCR machine (CFX Connect Real-Time PCR Detection System, Bio-Rad, Hercules, CA, USA) and iQ SYBR Green Supermix (Bio-Rad, Hercules, CA, USA) according to the guideline of manufacture. The reaction mixture (20 μL) contain 10 μL of iQ SYBR Green Supermix, 1 μL of cDNA template (100 ng), and 1 μL each of forward and reverse primers (Table S1) and 7 μL nuclease free water. RT-qPCR cycling began with 95 °C heat treatment for 10 min followed by 40 cycles of denaturation at 94 °C for 30 s, annealing at 55 °C temperature for 30 s, and extension at 72 °C for 20 s. Expression level of *EF1* as reference gene was used to normalize target gene expression levels^69^ under different treatments. PCR products were assessed by melting curve analysis. Quantitative analysis was performed using comparative CT (2^−ΔΔCT^) method^70^.

### RCH bioassay

RCH was measured according to a previous method^30^. The each developmental stage from eggs to adults was exposed to 5 °C for 6 h prior to − 10 °C for 1 h. For each treatment group, test individuals were placed in a Petri dish (10 × 15 mm). After 2 h of recovery at 25 °C after cold treatment, the survival rates of all developmental stages were determined. After gentle probing on the abdomen with a stick, autonomous movement of individuals was the criterion for being categorized as alive. Hatching in the 25 °C recovery state was used to assess egg survival. Adult emergence in the 25 °C recovery state was used to assess pupal survival.

### SCP measurement

SCPs were measured using a thermocouple (BTM-4208SD, LT Lutron, Taipei, Taiwan) to detect the release of the latent heat of fusion as body water froze, as described previously^32,71^. In SCP measurement, all developmental stages of FAW were examined after RCH treatment (exposed to 5 °C for 6 h prior to − 10 °C for 1 h). The thermocouples were kept in contact with the cuticle by putting the insect in a 1.5 mL tube and filling it with cotton wool to keep the insect and thermocouple together (Figure S1). They were then put in a styrofoam box (30 × 30 × 20 cm), and the box was placed into a freezer at − 80 °C. The cooling rate was measured as 1 °C min^−1^.

### Glycerol analysis in FAW hemolymph

The glycerol content of the samples was determined using the Glycerol Assay Kit (BioVision, Milpitas, CA, USA). We followed the manufacturer’s instruction for fluorometric measurements. In summary, a hemolymph from 10 fifth instar larvae (Day 1) was collected by cutting prologs of the treated larvae and mixed with a 100 µL volume of anticoagulant buffer (ACB). ACB was prepared with 186 mM NaCl, 17 mM Na_2_EDTA, and 41 mM citric acid^72^. The ACB was adjusted to pH 8.0 by the addition of NaOH. The resultant hemolymph was centrifuged at 13,500 rpm for 10 min at 4 °C. The supernatant (100 µL) were mixed with 100 µL of glycerol assay buffer (GAB) (provided by the kit) for 10 min on ice. The amount of 10 µL resulted supernatant (12,000 rpm, 5 min) was mixed with 86 µL GAB followed by 2 µL Probe (provided by the kit) and 2 µL glycerol enzyme mix (GEM) (provided by the kit) in a 96 well plate. The background control mixture was prepared as described above without GEM. In this assay, glycerol in the presence of glycerol enzyme mix is converted to an intermediate after incubation at 37 °C for 60 min, which reduces a colorless probe to a colored product with strong absorbance at 450 nm.

### Down-regulation of associated glycerol biosynthesis genes by RNA interference (RNAi)

RNAi was performed using dsRNA prepared with Megascript RNAi Kit (Ambion, Austin, TX, USA) according to the manufacturer’s instruction and a previous method^73^. Partial segments were amplified with gene-specific primers containing T7 promoter sequence at 5′ end (Table S1). dsRNAs (dsGPDH, dsGK1 and dsGK2) were synthesized at 37 °C for 4 h and then left at 70 °C for 5 min to inactivate T7 RNA polymerase. As control dsRNA (‘dsCON’), 300 bp fragment of enhanced green fluorescent protein (*EGFP*) was synthesized^74^. Three µg of dsRNA (1 µg/µL) was injected into each fifth instar larva with a Hamilton micro syringe. RNAi efficiency was determined by qPCR described above at 24, 48, 72, and 96 h post injection. For each treatment, at least 10 larvae were used. Each treatment was replicated three times.

### Sample preparation and HPLC condition

Hemolymph of 10 fifth instar (Day 1) was collected by cutting prologs of the treated larvae and mixed with a 100 µL volume of ACB. The resultant hemolymph was centrifuged at 13,500 rpm for 10 min at 4 °C. The supernatant (500 µL) was transferred to a new 1.5 mL tube, and then the same volume of acetonitrile (ACN) was added into the tubes and were shaken for 15 s. The tubes were incubated at room temperature for 10 min and then centrifuged as described above. The upper phase was collected in a new 1.5 mL tube. The previous step was repeated with the addition of 250 µL ACN to increase the purification. The final supernatant was filtered out by 0.22 µM syringe filters. The purified samples were directly used for HPLC in Metabolomics Research Center for Functional Materials, Kyungsung Univeristy (Busan, Korea). A reversed-phase HPLC connected to an evaporative light scattering detector (ELSD) (ELSD-LT II, Shimadzu, Japan) was optimized for simultaneous determination of cryoprotectant. HPLC separation was achieved using a Unison UK-Amino column (250 × 4.6 mm). Water and acetonitrile were used as the mobile phase. The ideal flow rate of 0.7 mL/min and a proportion of acetonitrile of 90% over 30 min were used to optimize the separation of cryoprotectant using isocratic elution conditions. The temperature of the ELSD detector was set at 30 °C with a temperature of column oven at 64 °C. Calibration curves were generated in GraphPad Prism by plotting the area against cryoprotectant concentration. Averages of the areas for each standard were calculated and plotted against the known concentrations.

### Data analysis

All studies were performed in three independent biological replicates. Results were plotted using Sigma plot 10.0. Means were compared by least squared difference (LSD) test of one-way analysis of variance (ANOVA) using PROC GLM of SAS program^75,76^ and discriminated at Type I error = 0.05.

## Supplementary Information

Supplementary Information.
