# Supplementary material for: Cold tolerance strategies of the fall armyworm, Spodoptera frugiperda (Smith) (Lepidoptera: Noctuidae)
Source: Sci Rep. 2022 Mar 8;12:4129. doi: 10.1038/s41598-022-08174-4 (PMC8904778; doi:10.1038/s41598-022-08174-4)
Supplement: Supplementary file 1 — Supplementary Information. [file 41598_2022_8174_MOESM1_ESM.docx]

**Cold tolerance strategies of the fall armyworm, *Spodoptera frugiperda* (Smith) (Lepidoptera: Noctuidae)**

**Mohammad Vatanparast, Youngjin Park^*^**

Plant Quarantine Technology center, Animal and Plant Quarantine Agency, Gimcheon 39660, Republic of Korea

^*^Corresponding author

Email) [parky1127@korea.kr](mailto:parky1127@korea.kr)

**Supplementary data**

**Figure S1.** The method has been used for supercooling point measuring.

**Figure S2.** The full-length gels of qPCR products.

**Table S1.** The primers were used in this study

**Table S2.** GenBank accession numbers used for alignment analysis and phylogenetic trees

**Figure S1.**


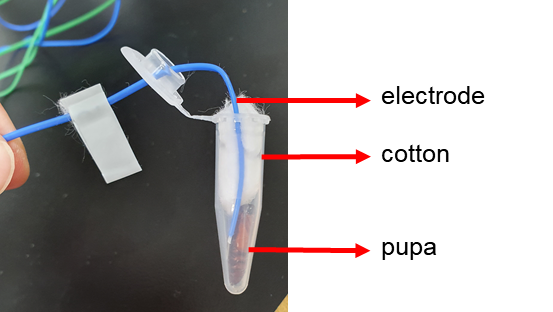


**Figure S2.**


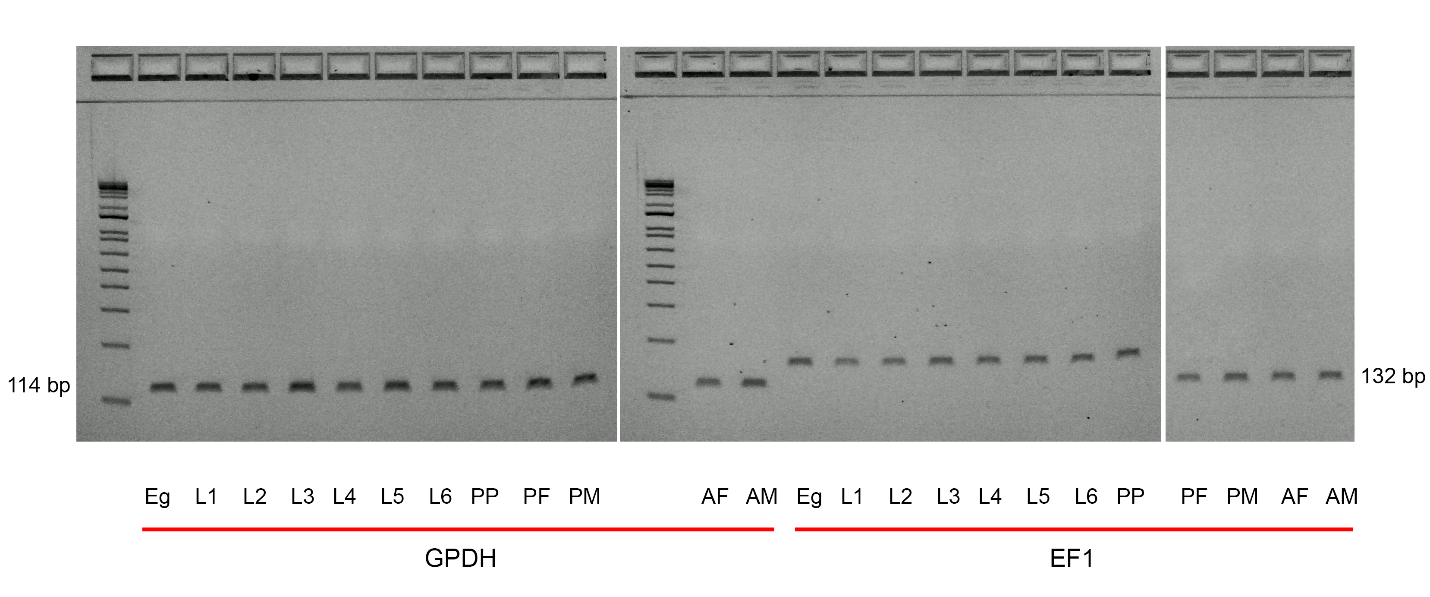


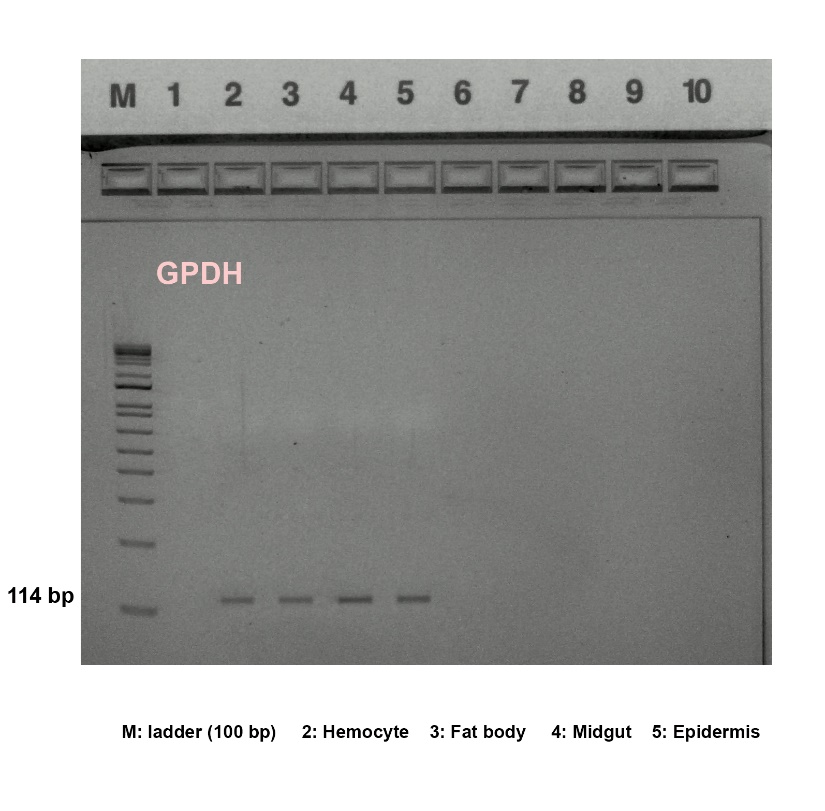


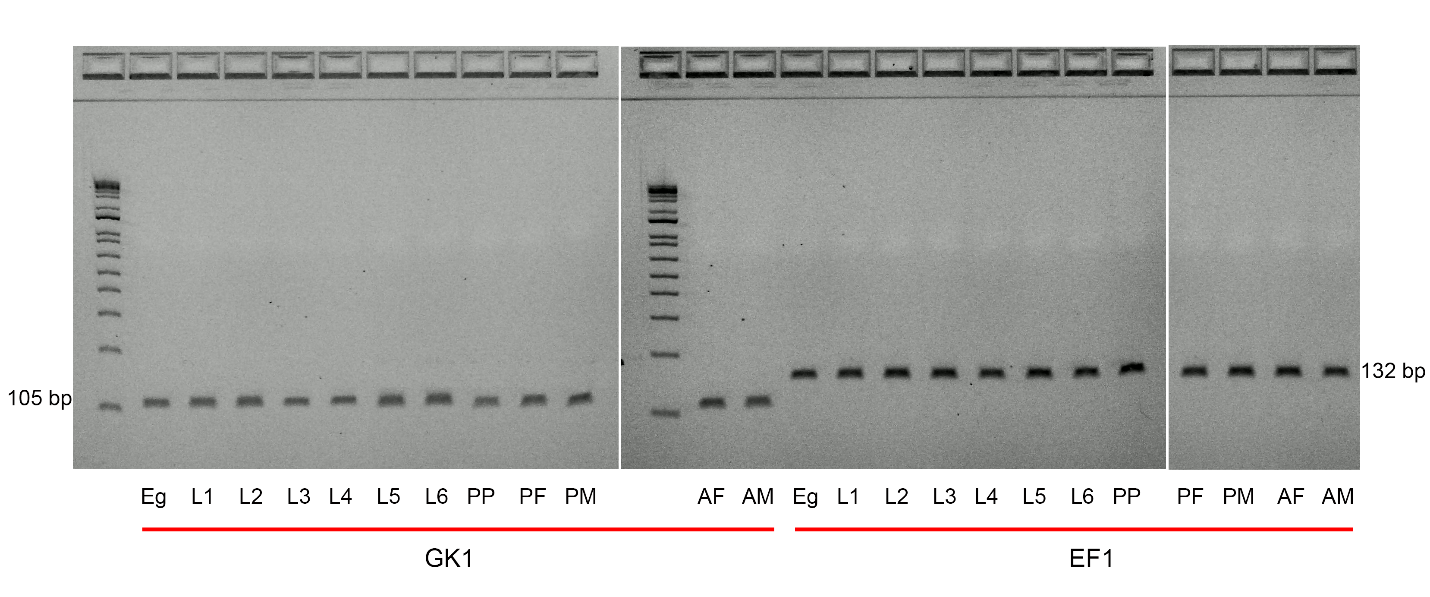


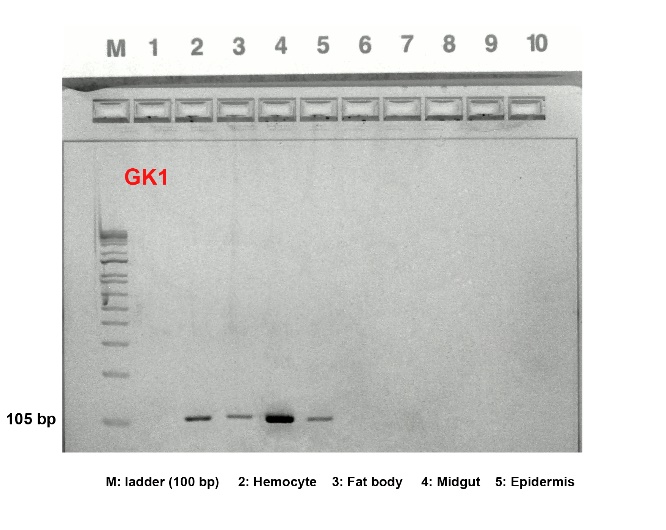


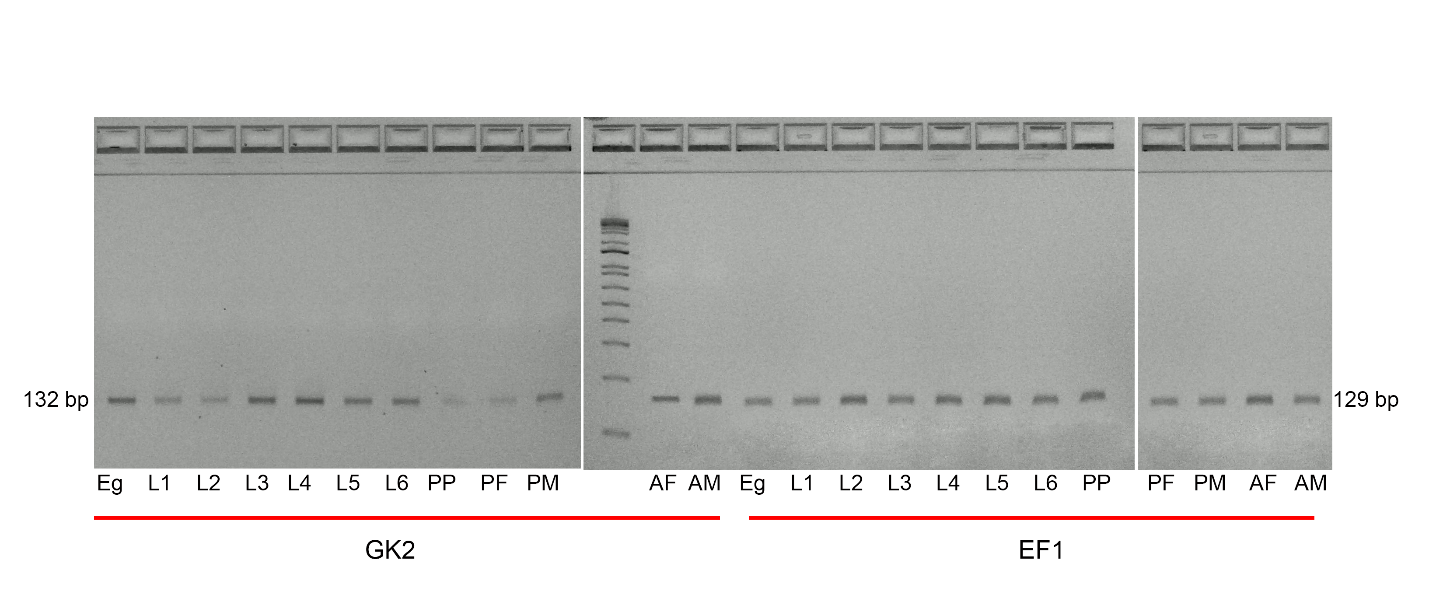


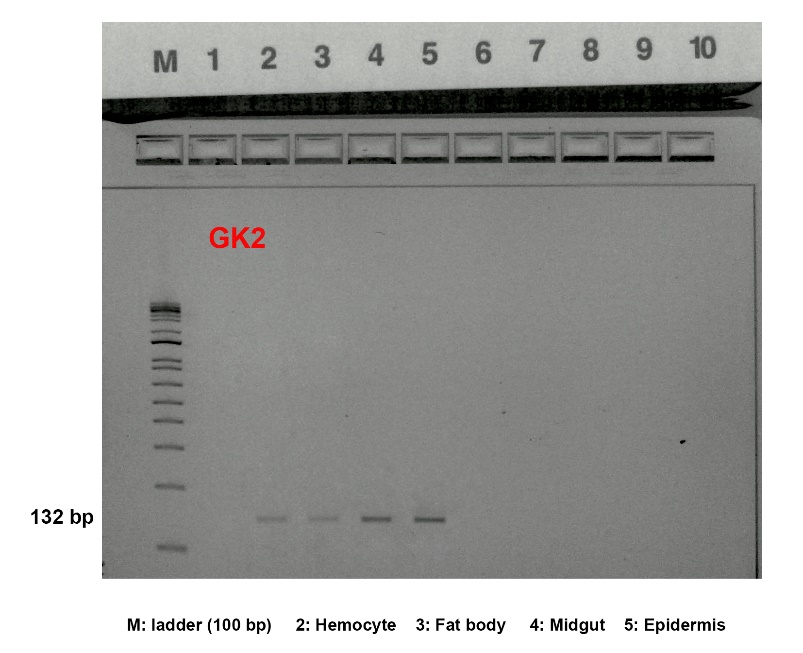


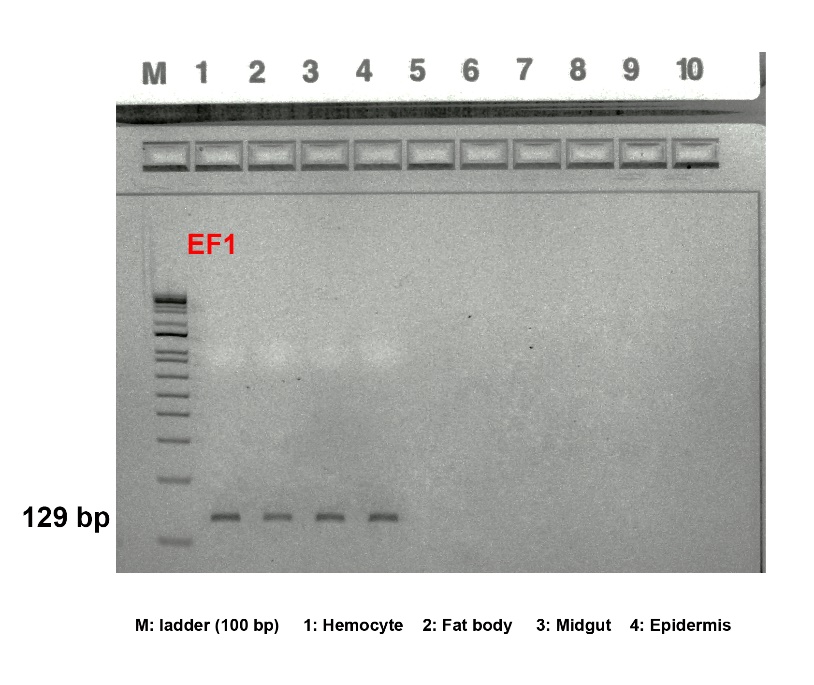
­

**Table S1.**

| Primer | Use | Orientation | Sequence (5ʹ - 3ʹ)* | Annealing  temperature (°C) | Amplicon  (bp) |
| --- | --- | --- | --- | --- | --- |
| Sf-GPDH | PCR | Forward  Reverse | TGCTGCTTGCACCTATGAT  CTCCTGTTCCTACCACCATAAC | 53 | 301 |
| Sf-GK1 | PCR | Forward  Reverse | CTCTCCTCATGAACCTGGAAAC CTACGGCTATGGATCCCTCTAA | 53 | 363 |
| Sf-GK2 | PCR | Forward  Reverse | CGTAGTCTGCAGTGGGATAAAG  CCCTTGTTGTGAGTGGATGAT | 53 | 267 |
| ds-GPDH | RNAi | Forward  Reverse | ^*^TAATACGACTCACTATAGGGAGATGCTGCTTGCACCTATGAT TAATACGACTCACTATAGGGAGACTCCTGTTCCTACCACCATAAC | 53 | 347 |
| ds-GK1 | RNAi | Forward  Reverse | TAATACGACTCACTATAGGGAGACTCTCCTCATGAACCTGGAAAC TAATACGACTCACTATAGGGAGACTACGGCTATGGATCCCTCTAA | 53 | 410 |
| ds-GK2 | RNAi | Forward  Reverse | TAATACGACTCACTATAGGGAGACGTAGTCTGCAGTGGGATAAAG TAATACGACTCACTATAGGGAGACCCTTGTTGTGAGTGGATGAT | 53 | 313 |
| dsEGFP | PCR | Forward  Reverse | TAATACGACTCACTATAGGGAGAGCTGACCCTGAAGTTCATCTG  TAATACGACTCACTATAGGGAGACACCTTGATGCCGTTCTTCT | 54 | 385 |
| qSf-GPDH | qPCR | Forward  Reverse | GTCTGAAGTGGCTGAAGAGAAA  ACCACGACCACTCTGAAGTA | 54 | 114 |
| qSf-GK1 | qPCR | Forward  Reverse | CGCTGGTTAAAGGACAATGTAAAG TCCACCGGTCATATTCCAAAC | 53 | 105 |
| qSf-GK2 | qPCR | Forward  Reverse | CGTAGTCTGCAGTGGGATAAAG AGCAATAGGTACGCCAAGTAAG | 53 | 132 |
| Sf-EF1 | PCR and qPCR | Forward  Reverse | TGGGCGTCAACAAAATGGA  TCTCCGTGCCAGCCAGAAAT | 53, 54 | 129 |

^*^T7 sequences were underlined.

**Table S2.**

| **Species** | **Abbreviation** | **Gene** | **Accession number** |
| --- | --- | --- | --- |
| *Acyrthosiphon pisum* | A.pis | glycerol-3-phosphate dehydrogenase | XP_008184495.1 |
| *Aedes aegypti* | A.aeg | glycerol-3-phosphate dehydrogenase | XP_021695848.1 |
| *Aedes albopictus* | A.alb | glycerol-3-phosphate dehydrogenase | XP_029726338.1 |
| *Agrilus planipennis* | A.pla | glycerol-3-phosphate dehydrogenase | XP_025832249.1 |
| *Anoplophora glabripennis* | A.gla | glycerol-3-phosphate dehydrogenase | XP_018575858.1 |
| *Aphis craccivora* | A.cra | glycerol-3-phosphate dehydrogenase | KAF0758096.1 |
| *Apis florea* | *A.flo* | glycerol-3-phosphate dehydrogenase | XP_012347457.1 |
| *Apis mellifera* | A.mel | glycerol-3-phosphate dehydrogenase | NP_001014994.1 |
| *Apis mellifera* | *A.mel* | glycerol-3-phosphate dehydrogenase | AAC14552.1 |
| *Bombus impatiens* | B.imp | glycerol-3-phosphate dehydrogenase | XP_012250275.1 |
| *Ceratitis capitata* | C.cap | glycerol-3-phosphate dehydrogenase | AAC32663.1 |
| *Diabrotica virgifera* | D.vir | glycerol-3-phosphate dehydrogenase | XP_028151611.1 |
| *Diuraphis noxia* | D.nox | glycerol-3-phosphate dehydrogenase | XP_015367828.1 |
| *Drosophila erecta* | D.ere | glycerol-3-phosphate dehydrogenase | EDV57920.1 |
| *Drosophila littoralis* | D.lit | glycerol-3-phosphate dehydrogenase | BAA34413.1 |
| *Drosophila melanogaster* | D.mel, *D. melanogaster* | glycerol-3-phosphate dehydrogenase | CAA32381.1 |
| *Drosophila yakuba* | D.yak | glycerol-3-phosphate dehydrogenase | XP_002089162.1 |
| *Galleria mellonella* | G.mel | glycerol-3-phosphate dehydrogenase | XP_026755779.1 |
| *Halyomorpha halys* | H.hal | glycerol-3-phosphate dehydrogenase | XP_014271686.1 |
| *Lasius niger* | L.nig | glycerol-3-phosphate dehydrogenase | KMQ85340.1 |
| *Leptinotarsa decemlineata* | L.dec | glycerol-3-phosphate dehydrogenase | XP_023016980.1 |
| *Locusta migratoria* | *L.migratoria* | glycerol-3-phosphate dehydrogenase | AAD05302.1 |
| *Ostrinia furnacalis* | O.fur | glycerol-3-phosphate dehydrogenase | XP_028171628.1 |
| *Papilio machaon* | P.mac | glycerol-3-phosphate dehydrogenase | KPJ08866.1 |
| *Papilio xuthus* | P.xut | glycerol-3-phosphate dehydrogenase | KPI91302.1 |
| *Photinus pyralis* | P.pyr | glycerol-3-phosphate dehydrogenase | XP_031358876.1 |
| *Plutella xylostella* | P.xyl | glycerol-3-phosphate dehydrogenase | XP_011557030.1 |
| *Riptortus pedestris* | R.ped | glycerol-3-phosphate dehydrogenase | BAN20845.1 |
| *Sitophilus oryzae* | S.ory | glycerol-3-phosphate dehydrogenase | XP_030754654.1 |
| *Trachymyrmex septentrionalis* | T.sep | glycerol-3-phosphate dehydrogenase | KYN44123.1 |
| *Triatoma infestans* | T.inf | glycerol-3-phosphate dehydrogenase | ABX56138.1 |
| *Tribolium castaneum* | T.cas, *T. castaneum* | glycerol-3-phosphate dehydrogenase | EEZ98249.2 |
| *Acyrthosiphon pisum* | A.pis | glycerol kinase | XP_016660307.1 |
| *Aedes aegypti* | A.aeg | glycerol kinase | XP_001651832.2 |
| *Agrilus planipennis* | A.pla | glycerol kinase | XP_025836773.1 |
| *Aphis craccivora* | A.cra | glycerol kinase | KAF0772382.1 |
| *Aphis gossypii* | A.gos | glycerol kinase | AJQ30111.1 |
| *Apis mellifera* | *A.mellifera* | glycerol kinase | XP_392723.3 |
| *Bactrocera dorsalis* | B.dor, *B. dorsalis* | glycerol kinase | XP_011201283.1 |
| *Bombus bifarius* | B.bif | glycerol kinase | XP_033306772.1 |
| *Bombus impatiens* | B.imp | glycerol kinase | XP_003485295.1 |
| *Bombus terrestris* | B.ter | glycerol kinase | XP_003394582.1 |
| *Bombyx mori* | *B.mori* | glycerol kinase | NP_001108335.1 |
| *Camponotus floridanus* | C.flo | glycerol kinase | XP_011252827.1 |
| *Cephus cinctus* | C.cin | glycerol kinase | *XP_015598250.1* |
| *Ceratitis capitata* | C.cap | glycerol kinase | XP_004525628.1 |
| *Dendroctonus ponderosae* | D.pon | glycerol kinase | XP_019760730.1 |
| *Diuraphis noxia* | D.nox | glycerol kinase | XP_015369586.1 |
| *Drosophila melanogaster* | D.mel | glycerol kinase | AAF47559.1 |
| *Drosophila miranda* | D.mir | glycerol kinase | XP_017138425.1 |
| *Drosophila mojavensis* | D.moj | glycerol kinase | XP_015017307.1 |
| *Harpegnathos saltator* | H.sal | glycerol kinase | XP_011139505.1 |
| *Helicoverpa armigera* | H.arm, *H. armigera* | glycerol kinase | XP_021192912.1 |
| *Heliothis virescens* | H.vir | glycerol kinase | PCG77980.1 |
| *Lasius niger* | L.nig | glycerol kinase | KMQ95878.1 |
| *Leptinotarsa decemlineata* | L.dec | glycerol kinase | KPJ09152.1 |
| *Manduca sexta* | M.sex | glycerol kinase | XP_030028496.1 |
| *Microplitis demolitor* | M.dem | glycerol kinase | XP_008546991.1 |
| *Musca domestica* | M.dom | glycerol kinase | XP_011296357.1 |
| *Myzus persicae* | M.per | glycerol kinase | XP_022172698.1 |
| *Nicrophorus vespilloides* | N.ves | glycerol kinase | XP_017771720.1 |
| *Ooceraea biroi* | O.bir | glycerol kinase | XP_011341937.1 |
| *Photinus pyralis* | P.pyr | glycerol kinase | XP_031356436.1 |
| *Pieris rapae* | P.rap | glycerol kinase | XP_022123608.1 |
| *Sitophilus oryzae* | S.ory | glycerol kinase | XP_030767742.1 |
| *Spodoptera litura* | S.lit, *S. litura* | glycerol kinase | XP_022832972.1 |
| *Tribolium castaneum* | T.cas, *T. castaneum* | glycerol kinase | XP_008190640.1 |
| *Trichoplusia ni* | T.ni | glycerol kinase | XP_026736616.1 |
| *Zerene cesonia* | Z.ces | glycerol kinase | XP_038211551.1 |
